# Supplementary material for: Fetal macrosomia, fetal insulin, and insulin-like growth factor- 1 among neonates in Lagos, Nigeria: A case-control study
Source: PLoS One. 2022 Aug 24;17(8):e0266314. doi: 10.1371/journal.pone.0266314 (PMC9401182; doi:10.1371/journal.pone.0266314)
Supplement: Attachment — Submitted filename: Letter of rebuttal_Final.doc [file pone.0266314.s002.doc]

6^th^ December 2021

The Editorial Board

PlosOne

Thank you for a timely review of the manuscript. The authors have reviewed the comments/ edits of the reviewers and have made changes as suggested by them. Where it was impossible to make the suggested review, we have provided reasons for our position. For ease of identification, we have provided files with tracked changes and clean copy. Kindly find below a point-by-point response to the reviewers’ comments.

When submitting your revision, we need you to address these additional requirements.

1. Please ensure that your manuscript meets PLOS ONE's style requirements, including those for file naming. The PLOS ONE style templates can be found at
   <https://journals.plos.org/plosone/s/file?id=wjVg/PLOSOne_formatting_sample_main_body.pdf> and 
   <https://journals.plos.org/plosone/s/file?id=ba62/PLOSOne_formatting_sample_title_authors_affiliations.pdf>

**Response**: We have ensured the compliance with the author’s guideline. However, if there we have omitted any requirement, it will promptly be attended to during production, if the manuscript is accepted for publication.

2. Please amend your current ethics statement to address the following concerns: Please explain why written consent was not obtained, how you recorded/documented participant consent, and if the ethics committees/IRBs approved this consent procedure.

**Response:** We obtained ethical approval for written consent, which was the consent procedure for this research. We have now indicated we obtained written consent in the manuscript as: “Informed written consent was obtained from the mothers. The ethics committee approved the consent and all other aspects of the research. There was however no potential harm to the mothers and babies as this was an observational study with no intervention. Autonomy and confidentiality was maintained throughout the study” line 169 – 173 (page 7)

Kindly note that informed written consent was obtained from the participants and was originally stated in the research dissertation at page 34 as published by the National Postgraduate Medical College of Nigeria. [1089-Article Text-6613-1-10-20190415 (3).pdf](../../../../../Library/Library/Containers/com.apple.mail/Data/Downloads/1089-Article%20Text-6613-1-10-20190415%20(3).pdf)

3. In your Data Availability statement, you have not specified where the minimal data set underlying the results described in your manuscript can be found. PLOS defines a study's minimal data set as the underlying data used to reach the conclusions drawn in the manuscript and any additional data required to replicate the reported study findings in their entirety. All PLOS journals require that the minimal data set be made fully available. For more information about our data policy, please see <http://journals.plos.org/plosone/s/data-availability>.

Upon re-submitting your revised manuscript, please upload your study’s minimal underlying data set as either Supporting Information files or to a stable, public repository and include the relevant URLs, DOIs, or accession numbers within your revised cover letter. For a list of acceptable repositories, please see [http://journals.plos.org/plosone/s/data-availability#loc-recommended-repositories](http://journals.plos.org/plosone/s/data-availability#loc-recommended-repositories). Any potentially identifying patient information must be fully anonymized.

Important: If there are ethical or legal restrictions to sharing your data publicly, please explain these restrictions in detail. Please see our guidelines for more information on what we consider unacceptable restrictions to publicly sharing data: [http://journals.plos.org/plosone/s/data-availability#loc-unacceptable-data-access-restrictions](http://journals.plos.org/plosone/s/data-availability#loc-unacceptable-data-access-restrictions). Note that it is not acceptable for the authors to be the sole named individuals responsible for ensuring data access.

We will update your Data Availability statement to reflect the information you provide in your cover letter.

**Response:** We have uploaded the data supporting our manuscript as supplementary file named S1.

3. Please amend the manuscript submission data (via Edit Submission) to include author Oluwakayode O Akinmola.

Response: This has been done. The correct name of Olukayode Akinmola has been added.

4. Please amend your authorship list in your manuscript file to include author Kayode Akinmola.

**Response**: This has been done. The correct name of Olukayode Akinmola has been added

5. We note you have included a table to which you do not refer in the text of your manuscript. Please ensure that you refer to Table 2 in your text; if accepted, production will need this reference to link the reader to the Table.

**Response:** This has now been done. Kindly refer to line 240, page 16.

[Note: HTML markup is below. Please do not edit.]

Reviewers' comments:

Reviewer's Responses to Questions

**Comments to the Author**

1. Is the manuscript technically sound, and do the data support the conclusions?

The manuscript must describe a technically sound piece of scientific research with data that supports the conclusions. Experiments must have been conducted rigorously, with appropriate controls, replication, and sample sizes. The conclusions must be drawn appropriately based on the data presented.

Reviewer #1: No

2. Has the statistical analysis been performed appropriately and rigorously?

Reviewer #1: Yes

3. Have the authors made all data underlying the findings in their manuscript fully available?

The [PLOS Data policy](http://www.plosone.org/static/policies.action#sharing) requires authors to make all data underlying the findings described in their manuscript fully available without restriction, with rare exception (please refer to the Data Availability Statement in the manuscript PDF file). The data should be provided as part of the manuscript or its supporting information, or deposited to a public repository. For example, in addition to summary statistics, the data points behind means, medians and variance measures should be available. If there are restrictions on publicly sharing data—e.g. participant privacy or use of data from a third party—those must be specified.

Reviewer #1: No

4. Is the manuscript presented in an intelligible fashion and written in standard English?

PLOS ONE does not copyedit accepted manuscripts, so the language in submitted articles must be clear, correct, and unambiguous. Any typographical or grammatical errors should be corrected at revision, so please note any specific errors here.

Reviewer #1: No

5. Review Comments to the Author

Please use the space provided to explain your answers to the questions above. You may also include additional comments for the author, including concerns about dual publication, research ethics, or publication ethics. (Please upload your review as an attachment if it exceeds 20,000 characters)

Reviewer #1: General comments
It is difficult to understand at the time of reading what the rationale of the study is and why it seems so important in the Nigerian population to know if there is an association between fetal insulin and IGF1 levels and macrosomia.

**Response:** We have recast the manuscript and provided further study justification. We stated that the two main pathways of influence of fetal weight was fetoplacental and genetic factors. One of the feto-placental factors is the elaboration of insulin and IGF-1. In most paragraphs of the introduction section, the study justification was presented. Specifically, and in line with the reviewer’s comment, we stated:

“Establishing an association between IGF-1, insulin and macrosomia among Nigerian babies can aid further interventional research on the feto-maternal pathophysiology of macrosomia and its attendant immediate maternal complications at birth and later life associated disease risks reductions” line 123- 126 (Page 5)

What clinical impact do the authors expect from their findings, if any?

Moreover, there is a discrepancy between the announced objective (relationship between fetal insulin, IGF1 and macrosomia) and the results and the conclusion which essentially concerns the maternal, neonatal and biological clinical predictive factors of macrosomia.

**Response:** The authors did not see any discrepancies between the research objectives, results and conclusion. We reported on our primary objective of the association between IGF-1 and macrosomia. We further conducted multivariable regression modelling to assess the association between IGF-1 and macrosomia. We then reported other secondary findings.

Why is the study and the collected samples from 2015 and only submitted for publication in 2021?
Response: the observed interval between research and publication is correct. We believe in transparent scientific reporting, which was why the dates are as reported. Essentially, the study was conducted in part fulfilment for the requirement of the award of the Fellowship of the National Postgraduate Medical College of Nigeria. It is being reported in a scientific journal now because the candidate’s mentors have encouraged him to disseminate his research findings in a peer-reviewed journal.

Specific comments
MM
Justify the choice of a 2/1 control case study?

R**esponse:** Case control studies might have case: control ratio of 1:1, 1:2, 1:3, 1:4 or vice versa. We adopted a 2:1 ratio by recruiting 100 cases and 50 control to minimize the challenges of enrolment of controls, and to reduce the study duration and costs. Furthermore, some sub-analyses were conducted among the cases. Thus, a larger sample of the cases will improve the conclusion of such intraclass analyses.

Why did you not match the controls on BMI and parity, which are major confounding factors?

**Response:** Known confounders in research may be handled during participants recruitment and data analysis. Due to the difficulty of matching using more than one variable, matching was done with only one variable. However, we utilized multivariable regression modelling to correct for other known confounding variables, including parity. We have now added parity as a variable in the multivariable model (Table 2)

what is the rationale for the evaluation of neonatal anthropometric measurements of the choice of occipitofrontal circumference ? It does not seem that it is a representation of the neonatal fat mass.

**Response:** We thank you for this question. We decided to show all the anthropometric measurements, including the occipitofrontal circumference. Although, at the moment OFC does not have biologic direct correlation with the neonatal fat mass, we felt that showing all the parameters might be valuable to the literature in future. If the reviewers and indeed the editor strongly feel otherwise, the authors will remove OFC as an anthropometric measure.

What is Digital weighing scale ?

**Response**: The weighing scale used for participants weight measurement has been further described. Line 147 - 148

The definition of macrosomia is not given and if it is a question of babies weighing more than 4000g as it seems to appear in the results, then some babies are not macrosome since this depends on the gestational age at birth and therefore on the weight curves at birth. Indeed at term 4000kg is not a fetus > 90th percentile and is not considered as macrosomic newborn. Please give the definition of macrosomia and justify this choice for the interest of the study.

**Response:** For this study, the definition of macrosomia is birth weight weight ≥ 4000g. This has been defined in the introduction (line 102 - 102) and methodology (line 138) with references. Also, we used an absolute weight of 4000g at birth as it is more pragmatic and will avoid the use of different cut-off weights for different neonates.

Table 1 should be simplified and fit on one page.

**Response:** The Table has been re-designed to fit 2-pages in Landscape format as it was not possible to make it a page.

On the other hand, as indicated, the groups between macrocosmic and non-macrocosmic fetuses are not comparable and BMI and parity appear as confounding factors and limit the interpretation of the results.

R**esponse:** Table 1 is a bivariate analysis. We corrected for the confounding variables in Table 2. Table 2 showed the univariable and multivariable analysis that corrected for BMI and parity. We re-analyzed the multivariable model by including parity in the model. This did not change the point estimates or direction of association of the relationship between insulin and the outcome.

Results
Line 204 It is not indicated either in MM or in the results which confounding factors were entered in the logistic regression model. Please indicate these details

**Response:** Thank you for this observation. This has now been stated in the methods (line 192 - 194) and as legend of Table 2

When interpreting the results in Table 2, the sentence maternal BMI at birth (P205
value =0.003), fetal gender (P-value < 0.001), fetal cord serum PGF-1 (< 0.001), and insulin
206 assay (P-value =0.027) were the statistically significant predictors of fetal macrosomia should be changed to "were significantly associated of ..." This is not a prediction model

**Response**: This has been corrected (Line 237 -238)

Similarly, in the conclusion, the word prediction should be changed to association.

**Response:** This has been corrected in the conclusion of the abstract and the conclusion after discussion (Line 331 -332)

Discussion
It seems when reading the discussion that it has been reported in non-diabetic macrocosmic fetuses an increase in insulin and IGF1 and this study just reports the same results but in a Nigerian population. I have difficulty understanding the clinical impact of these results and this is not at all discussed. Metabolic consequences in the macrosomic newborn? Particular follow-up of these children?
There are many points to review in the objective, methodology and results in order to better understand the message that the authors wish to give in this article

**Response:** This is a basic science research that provides the biochemical basis for fetal macrosomia, a relative common pregnancy complication in Nigeria. On Page 20, lines 308 - 315 of the revised manuscript, the authors have included clinical implications of the study findings, including a potential use of prenatal assay of fetal IGF-1 and insulin to predict fetuses who might develop fetal macrosomia.

6. PLOS authors have the option to publish the peer review history of their article ([what does this mean?](https://journals.plos.org/plosone/s/editorial-and-peer-review-process#loc-peer-review-history)). If published, this will include your full peer review and any attached files.


If you choose “no”, your identity will remain anonymous but your review may still be made public.


**Do you want your identity to be public for this peer review?** For information about this choice, including consent withdrawal, please see our [Privacy Policy](https://www.plos.org/privacy-policy).

Reviewer #1: No

[NOTE: If reviewer comments were submitted as an attachment file, they will be attached to this email and accessible via the submission site. Please log into your account, locate the manuscript record, and check for the action link "View Attachments". If this link does not appear, there are no attachment files.]

While revising your submission, please upload your figure files to the Preflight Analysis and Conversion Engine (PACE) digital diagnostic tool, <https://pacev2.apexcovantage.com/>. PACE helps ensure that figures meet PLOS requirements. To use PACE, you must first register as a user. Registration is free. Then, login and navigate to the UPLOAD tab, where you will find detailed instructions on how to use the tool. If you encounter any issues or have any questions when using PACE, please email PLOS at [figures@plos.org](mailto:figures@plos.org). Please note that Supporting Information files do not need this step.
