# Supplementary material for: Fetal macrosomia, fetal insulin, and insulin-like growth factor- 1 among neonates in Lagos, Nigeria: A case-control study
Source: PLoS One. 2022 Aug 24;17(8):e0266314. doi: 10.1371/journal.pone.0266314 (PMC9401182; doi:10.1371/journal.pone.0266314)
Supplement: Attachment — Submitted filename: Response to Reviewers.docx [file pone.0266314.s003.docx]

**Editor’s comment:**

The aim of the present manuscript by Akinmola et al was to measure fetal insulin and insulin-like growth factor-1 (IGF-1) levels in macrosomic fetuses delivered at term in Lagos State, Nigeria. Overall, the research was aimed at determining potential correlations between both hormones with fetal anthropometric variables. Authors provide evidence that maternal BMI at birth, neonate’s gender, and fetal cord serum IGF-1 and insulin were correlated with fetal macrosomia. Authors emphasize that this association might help identify these hormones as important predictive factors to prevent macrosomia. Hence, the paper might bear important clinical relevance.

The present manuscript constitutes a revised version of the paper. Authors have addressed reviewer’s concerns in a satisfactory manner. In particular, authors expand on the rationale of the study and better elaborate the justification for the study. In addition, authors clarify a number of methodological issues in the revised version.

In summary, the present manuscript is a concise and straightforward analysis of the link between neonate insulin, IGF-1 and macrosomia. While mainly of a descriptive nature, the paper includes valuable informmation.

**Authors’ response**: We are grateful for the favourable and fair assessment of our manuscript

**Editor’s comment:**

Minor points:
Lines 50, 203, 207, 238, 300, : should be IGF-I, not PGF-1.

**Authors’ response**:

We have changed PGF-1 to IGF-1 throughout the manuscript. We further utilized the “find” in Microsoft word to ensure all PGF-1 has been changed to IGF-1.
